# Impact of the COVID-19 pandemic on intimate partner violence in Sudan, Malawi and Kenya

**DOI:** 10.1186/s12978-021-01272-y

**Published:** 2021-11-07

**Authors:** Salma A. E. Ahmed, Josephine Changole, Cynthia Khamala Wangamati

**Affiliations:** 1 Mother and Child Health Directorate, Sudan Federal Ministry of Health, P.O. Box 303, Khartoum, Sudan; 2 Blantyre, Malawi; 3 Department of Community Medicine and Global Health, Institute of Health and Society, University of Oslo, Blindern, P.O. Box 1130, 0318 Oslo, Norway

**Keywords:** COVID-19, Intimate partner violence, Legal environment, Health systems response, Sudan, Malawi, Kenya

## Abstract

The COVID-19 infection control and prevention measures have contributed to the increase in incidence of intimate partner violence (IPV) and negatively impacted access to health and legal systems. The purpose of this commentary is to highlight the legal context in relation to IPV, and impact of COVID-19 on IPV survivors and IPV prevention and response services in Kenya, Malawi, and Sudan. Whereas Kenya and Malawi have ratified the Convention on Elimination of all forms of Discrimination against Women (CEDAW) and have laws against IPV, Sudan has yet to ratify the convention and lacks laws against IPV. Survivors of IPV in Kenya, Malawi and Sudan have limited access to quality health care, legal and psychosocial support services due to COVID-19 infection control and prevention measures. The existence of laws in Kenya and Malawi, which have culminated into establishment of IPV services, allows a sizable portion of the population to access IPV services in the pandemic period albeit sub-optimal. The lack of laws in Sudan means that IPV services are hardly available and as such, a minimal proportion of the population can access services. Civil society’s push in Kenya has led to prioritisation of IPV services. Thus, a vibrant civil society, committed governments and favourable IPV laws, can lead to better IPV services during the COVID-19 pandemic period.

## Introduction

Health experts advise populations to stay at home as a COVID-19 prevention measure. However, home may not be the safest place for intimate partner violence (IPV) survivors as they are confined in the same spaces with their abusers. The purpose of this commentary is to highlight the health and the legal contexts in relation to IPV, and the impact of COVID-19 on IPV survivors and IPV prevention and response services in Kenya, Malawi, and Sudan. Lessons can be drawn across the countries from their response and be applied across different settings in sub-Saharan Africa.

Globally, one in three women has experienced either physical and/or sexual intimate partner violence or non-partner sexual violence in their lifetime [1]. It is estimated that the prevalence for IPV in sub-Sahara Africa is 35.5% [2]. According to the 2014 Kenya Demographic and Health Survey, 39% of ever-married women and 9% of men aged 15–49 report having experienced spousal physical or sexual violence [3]. A similar survey in Malawi, indicated that 42% of ever-married women have experienced spousal violence; the most common type of spousal violence is emotional violence (30%), followed by physical violence (26%) and sexual violence (19%) [4]. In Sudan, there are no national figures on IPV prevalence. However, we postulate that the prevalence is high as a study of women in the eastern states of Sudan estimated that 33.5% had experienced physical and sexual abuse from a current or former partner [5]. In countries like Sudan, Malawi and Kenya, IPV is often under-reported due to normalization of violence against women, the stigma associated with reporting, weak laws and poor enforcement of laws, and non-existent or poor response systems [6, 7]. Intimate partner violence has negative physical and mental health consequences. They include but are not limited to physical injury, chronic pain, unwanted pregnancies, sexually transmitted diseases, anxiety, depression, and post-traumatic stress disorder, miscarriages and poor child development [8].

## Legal environment related to intimate partner violence

Kenya and Malawi ratified the CEDAW convention in 1984 and 1987, respectively. Consequently, efforts have been put in place to have to ensure that rights of women are not infringed upon. Kenya has several laws against IPV. The Kenyan Constitution of 2010 states that every person has right to freedom and security of their person which includes the right not to be subjected to any form of violence from either public or private sources, any form of torture whether physical or psychological or cruel, inhuman or degrading treatment [9]. The Sexual Offenses Act, 2006 stipulates prevention and protection of all persons from harm from sexual acts and access to justice and psychosocial support, however, marital rape is not criminalised [9]. The Land Act, 2012 gives women rights to matrimonial property while The Land Registration Act, 2012 provides for spousal consent in any dealing with matrimonial property [9]. The Matrimonial Property Act, 2013 outlines the rights and responsibilities of spouses in relation to matrimonial property [9]. The Marriage Act, 2014 stipulates the minimum age in marriage (18 years) and types of marriages and guarantees parties to a marriage, equal rights at the time of the marriage, during the marriage and at the dissolution of the marriage [9]. The Protection against Domestic Violence Act, 2015 outlines the protection and relief of members of a family from domestic violence [9].

In keeping with its constitutional and international human rights obligations, the Malawi Government enacted seven gender related laws whose purpose was to ensure the commitment of the State to eliminate gender-based violence occurring within a domestic relationship, and to provide for effective legal remedies and other social services to persons affected by domestic violence [10]. The laws prohibit discrimination against women, promote gender equality, protect children from economic exploitation or any treatment, work or punishment that may be hazardous or harmful to their education, health or to their physical, mental or spiritual or social development, discrimination in work, business and public affairs; and deprivation of property, including property obtained by inheritance (Chapter IV, Sections 20, 22, 23, and 24 of the Constitution) [11]. These laws include: Prevention of Domestic Violence Act, 2006 [12]; the Child Care, Protection and Justice Act, 2010 [13]; the Deceased Estates (Wills, Inheritance and Protection) Act, 2011 [14]; the Gender Equality Act, 2012 [15], the Marriage, Divorce and Family Relations Act, 1014 [16], and the Trafficking in Persons Act, 2015 [17]. It should be noted that rape is also not criminalised in Malawi.

Sudan is yet to ratify the CEDAW [7]. There are no laws stipulating punishment for the IPV perpetrators, legal aid and psychosocial support for survivors [7, 18]. Moreover, marital rape is not recognised in the Sudanese Criminal law [19]. The family law in Sudan stipulates the obedience of the wife to her husband including that she cannot deny him sexual intercourse as long as he pays her *Nafaga* (financial support) [19]. The concept of *Qawama* (male guardianship) in Sudanese laws based on Sharia gives the husband the right to make decisions on the woman’s life, restrict her movement and deny access to health and legal services [18].

Despite the existence or non-existence of laws, Sudan, Malawi, and Kenya are patriarchal societies where women and men are socialised to be different, with men allocated dominant and public roles and women submissive and domestic roles [20]. Marital customs relegate the duty of child rearing and domestic work to women and assign men the role of provision, thereby creating power differences as men assume dominant responsibilities whereas women get submissive ones [21]. These gender inequalities are responsible for weak enforcement of gender-based violence (GBV) laws in Kenya and Malawi, along with the lack of GBV laws in Sudan, and the lack of a well-resourced functional system to address needs and concerns of IPV survivors as well as prevent IPV in all the three countries as violence against women and girls has been normalised.

## Health, legal and psychosocial services offered to IPV survivors

Comprehensive GBV prevention and response services are existent in Kenya but they are limited and mostly found in urban and peri-urban areas; response services are almost non-existent in rural areas [22]. In these countries, most health facilities are understaffed, and lack clinical guidelines, equipment, and medical supplies to offer quality health services to IPV survivors [22]. Moreover, most health providers lack training on how to manage IPV survivors, are poorly remunerated, and overworked and as such unmotivated [22]. Similar issues have been reported in Malawi [23]. In Kenya, gender desk services have been established to provide legal services to IPV survivors. However, it is reported that when GBV survivors report violence incidents to the police, some police officers’ side with the perpetrators (due to bribery and normalization of violence) and either send them away or victimize them further [22, 24, 25]. When cases of violence are taken to court by the police, the judiciary has a backlog of cases and as such, processes are lengthy forcing the survivor to bear transportation costs to cover for the numerous court adjournments [25]. Safe houses are few and usually crowded, and in most cases non-existed [25]. In Malawi, Police Victim Support Units were established in all districts in response to, and to prevent all forms of gender-based violence [26]. However, it is difficult for survivors of IPV to get justice due to limited capacity of the police.

According to a recent assessment in Khartoum, Sudan, the services to IPV survivors are mostly provided by civil society organizations and they are poorly integrated, have lengthy referral processes and their quality is questionable [27]. For instance, the helpline is rarely operated by a delegated trained cadre who is capable of dealing with survivors first-hand [27]. In addition, shelter for survivors is not part of the service package provided, however, some care providers can act informally to help survivors with temporary accommodation which poses risk to both parties and not suitable as a long-term measure [27]. After three decades of dictator Islamist rule which was overthrown in August 2018, women issues have been heavily politicized [28]. As such, the legal and health systems’ response to IPV has been sub-optimal as IPV is often unrecognized as a health right and a human rights violation. Consequently, the response to IPV lacks political commitment, is poorly funded, and unorganized [7]. In Sudan, most women do not know their rights and legal issues related to IPV, for instance, the ability to file a case for documentation purposes without going to court and pressing charges [27].

## Intimate partner violence during COVID-19 pandemic “the shadow pandemic”

Intimate partner violence is currently regarded as the shadow pandemic as the incidence rates have increased during COVID-19 pandemic [18, 29, 30]. Since the outbreak of COVID-19, emerging reports from those on the front line have shown that violence against women, particularly domestic violence, has intensified. In Kenya, the National Council for the Administration of Justice reported that sexual offences during the first quarter of 2020 increased by 35.8%. Risk factors for violence were more pronounced for women and other vulnerable populations [31]. Moreover, police statistics indicate that the number of GBV cases recorded between January and June 2020 increased by 92.2% compared with those between January and December 2019 [32].The same report established that 71.0% of the 2416 cases of GBV reported between January and June, 2020 were female victims with the main perpetrators being youthful males aged 18–33 years who are in a family and/or intimate partner relationship context [32]. In Malawi, some non-governmental organisations reported an increase in GBV cases during the lockdown as a COVID 19 containment measure, however, there are no definite figures [33].

The IPV increase in households is perceived to be caused by the stress directly or indirectly caused by COVID-19 infection control and prevention measures [34]. Stressors include loss of employment which results into financial hardship and prevention measures such as movement restrictions [34]. In addition, disruption of support mechanisms during the pandemic may exacerbate IPV consequences and limit women access to help [35]. It is expected that IPV and its consequences to be higher among other groups such as lesbian, gay, bisexual, and transgender (LGBT) individuals due to social stigma and legal status of those groups in Sudan, Malawi and Kenya.

## Impact of COVID-19 on health and legal systems response to intimate partner violence

Stay at Home directive aimed at prevention and containment of the coronavirus spread has been applied without cognizance of existing risks to vulnerable groups who face restricted movement exposing them to violence, inequalities, and stifling voices of survivors of violence and abuse [36]. The inability and reduced access to income-earning opportunities, loss of jobs, and livelihoods have exacerbated gender-based violence. The limited access to service providers such as health facilities, police stations, and access to courts due to physical distancing and curfew measures have hampered redress to affected violence survivors [36].

## Kenyan government response

Kenya confirmed its first COVID-19 case on March 13, 2020. In response to the gradually increasing numbers of confirmed cases, the Government of Kenya took proactive action and ordered the closure of Kenya’s international airports, introduced a nightly curfew, closed schools, and recommended that those who can work from home do so to observe principles of physical distancing [37]. These measures aim to reduce citizens’ and residents’ mobility to slow transmission of the virus and prevent an overwhelming burden on the healthcare system. While these interventions slowed the coronavirus’s spread to some degree, they brought with them a multitude of associated social and health issues and negatively affected families [37].

On the 28 February 2020, an Executive Order No. 2 of 2020 established the National Emergency Committee on Coronavirus; the Committee lacked representation from the Ministry of Public Services and Gender neglecting gender dimensions of COVID-19 in the response plan. In April 2020, the Ministry of Health (MOH) established a Community Engagement Health Strategy to respond to COVID-19; however, the strategy did not address GBV. Following a push from the civil society on the need to address GBV, the MOH released guidelines that deemed health care needed by GBV survivors as essential in May 2020 which lacked clear sector-specific guidelines on the full range of comprehensive services and programmes for GBV survivors [22].

Essential services such as medical and psychosocial services, police and judicial services, shelters and other social services, and community-based prevention work were disrupted in the initial phases of the pandemic. Survivors were turned away from police stations or asked to return later. The national GBV hotline limited its operating hours due to curfew restrictions hindering survivors from reporting incidences of GBV. Other factors that hindered access to GBV services included limited transportation services, fear of police mistreatment and contracting COVID-19 in public spaces, among others. In June 2020, the National Police Service set up a toll-free hotline for survivors to report GBV. The police also utilised social media platforms to increase awareness and to promote the use of their hotline. In August 2020, the police launched PoliCare, a one-stop model police station, where survivors can access critical multi-sectoral services; these services are only available to residents of Nairobi City [22].

The courts were closed in the initial phases of the pandemic. On reopening, they held fewer sessions and issued lenient bail to GBV perpetrators to avoid remand prison overcrowding. Some courts closed due to staff contracting COVID-19. The courts only handled child sexual abuse and rape cases termed as essential services, and all other GBV cases were handled at the police station level. It was perceived that the slower operation of the courts might have led to witness tampering and repeat offences. From July 2020 onwards, courts made provisions for electronic filing of cases and set up virtual court hearings to comply with social distancing guidelines. However, these services were only available to middle class survivors who could access and afford internet services [22].

Despite emergency clinical and post-rape care services being deemed essential in May 2020 by the MOH, GBV services were inaccessible due to health facilities being converted into quarantine centres, and health care providers being redeployed to COVID-19 quarantine and isolation centres. GBV survivors also feared visiting health facilities due to fear of contracting COVID-19; or of being forced to test for COVID-19 and being made to quarantine on testing positive. Due to COVID-19 restrictions, organisations providing psychosocial services to survivors closed operations in the initial phases of the pandemic. However, lobbying by civil society due to increase in GBV cases led to additional resources by foreign donors to hire counsellors, advertise services provided by the GBV hotline, and strengthening of referrals to survivors. Other service providers resumed operations and set up toll-free hotlines, phone calls, and other virtual platforms to offer counselling services to survivors. However, these services were elitist as they could only be accessed by middle class women. To address this limitation, some organisations have trained and utilised community health volunteers to support women and girls in their communities to offer psychological first aid [22].

Early days of the pandemic were characterised by an increased demand for scarce shelter services and closure of several shelters. The few that remained open were reluctant to admit more people due to limited food and non-food items and demanded a COVID-19 medical certificate, which was costly to obtain. The government has mapped out existing and new shelters shared information with GBV actors as a move towards addressing existing gaps; one of the counties (Makueni County) has established a fully resourced shelter. In the initial phases of the COVID-19 pandemic, all community-based prevention and awareness-raising activities were halted. In May, the MOH drafted guidelines on resuming prevention work and awareness raising activities communities. These enabled organisations to resume in-person contact with communities. However, community health workers lacked personal protective equipment putting them and survivors risk for COVID-19 [22].

### Malawi government response

The first three cases were confirmed on 2nd April 2020 [38], cases rose with the repatriation of citizens from South Africa [39]. Although the country seemed to have been spared in the first wave, the second wave was so devastating with over 500 cases reported per day [40]. Currently, reported cases have gone down to as low as 20 cases per day [38]. In response to the pandemic, the Malawi government declared a state of emergency and announced a 21-day lockdown even before the country had reported any case, only to be cancelled [41]. In essence, the country has never had a lockdown [40]. However, the government put down measures to prevent the spread of the virus such as school closures, a night-time curfew, limited number of people in public transport, mandatory mask up in groups and no gatherings over 50 people [39, 42].

In Malawi, Police Victim Support Units were established in all districts in response to, and to prevent all forms of gender-based violence [26]. Malawi have established a hotline to support IPV survivors. However, it was difficult for survivors of IPV to access IPV prevention and response services due to limited capacity of relevant service providers. The situation has been exacerbated due to the shift of focus and limited resources to the COVID-19 pandemic. Police officers and health care personnel have been deployed to quarantine camps, straining the already constrained human resources [43]. Sexual and reproductive health workers have been reallocated to the COVID-19 response [43]. The capacity of courts to process GBV criminal cases has been reduced because of changes in operational protocols asking judicial staff to work in shifts. The police response to IPV cases was limited due to poor supply of personal protective equipment (PPE) which created fear of COVID-19 infection [43]. Victim Support Unit (VSU) staff working are also working in shifts despite the high demand for services [43]. Travel restrictions barred some women from IPV prevention and response services. Restrictions on movement and the closure of communal spaces such as markets or youth-friendly centres closed important spaces where IPV survivors received information about relevant services [43]. Furthermore, due to the recommended social distancing, public transport fares have doubled, making it impossible for most survivors to seek help [44]. Additionally, fear of getting infected with COVID-19 detered IPV survivors from accessing health care services [43].

### Sudan government response

The first COVID-19 case in Sudan was reported on March 13, 2020, soon after, the Sudanese government declared a national emergency. Accordingly, learning institutions were closed and all mass gatherings were banned followed by closure of international borders [45]. However, a reprieve in late March to allow Sudanese travellers in the country is perceived to have led to importation of more COVID-19 cases [45]. Consequently, dusk-to-dawn curfews in Khartoum, the capital city, were enforced from April 18, 2020 with movement restrictions extended to other parts of the country [45].

As part of the national response to COVID-19 pandemic, the Ministry of Social Welfare (MoSW) in Sudan has established a hotline to support IPV survivors [7, 46]. Since these services are quite new (before the pandemic IPV services were non-existent), survivors were hesitant to seek health services as they doubted their quality. Moreover, most of the targeted populations were unaware of the hotline existence because it was advertised mostly on social media platforms in a population that has limited access to internet. The available IPV services were not comprehensive as guidelines on prevention and response are non-existent, and there was a lack of psychosocial and legal aid support. The unitoperating the hotline at the MoSW mainly coordinated and referred survivors to service providers which made services fragmented, inefficient, and less sustainable [27]. Inadequacy of services and lengthy procedures due to lack of integration and coordination between different sectors jeopardized the quality of services provided to victims and survivors of IPV and that could prevent them from seeking future services [27].

## Conclusion

COVID-19 has exacerbated IPV in Kenya, Malawi, and Sudan because of recommended pandemic control and prevention measures. Kenya and Malawi have systems in place to address IPV; although services are sub-optimal, they are accessed by a sizeable proportion of the population. COVID-19 infection control and prevention measures such as re-allocation of health providers COVID-19, IPV prevention and response services being provided in shifts, closure of courts and/or digitalizing services have limited access in times of great demand due to increased cases in Kenya and Malawi.

Kenya only included GBV in the COVID-19 response plans after an outcry from civil society; an indication that civil society is an important voice in IPV prevention and response in societies that are patriarchal and do not prioritize gender issues more so in the pandemic period.

All the countries had helplines to support IPV survivors. However, these services were only accessible to a marginal section of the population. Kenya has shown that trained community health workers can offer IPV prevention and control services to survivors to marginalized groups that have no access to the internet or phone services during the COVID-19 infection control and prevention measures period. The challenge remains in equipping community health workers with personal protective equipment, transport facilitation and wages.

Sudan does not have a prevention and response plan which can be attributed to lack of political will and a legal framework to back the establishment of the services. Laws play an important role in supporting establishment of IPV legal and health services despite provision of sub-optimal services as indicated in the case of Kenya and Malawi. There is a need for advocacy for legal reform in Sudan to address issues related to IPV in laws and legal frameworks including criminalization of marital rape and prioritization of provision of health and legal services to IPV survivors.

## Data Availability

Not applicable.

## Abbreviations

CEDAW: Convention on Elimination of all forms of Discrimination against Women
COVID-19: Coronavirus disease
GBV: Gender-based violence
IPV: Intimate partner violence
LGBT: Lesbian, gay, bisexual, and transgender
MoSW: Ministry of Social Welfare
SSA: Sub-Sahara Africa
VSU: Victim Support Unit
